# A Case of Symptomatic Paroxysmal Complete Atrioventricular Block

**DOI:** 10.7759/cureus.33271

**Published:** 2023-01-02

**Authors:** Bradley Casey, Amol Bahekar, Divyang Patel, Raviteja Guddeti

**Affiliations:** 1 Internal Medicine, Cape Fear Valley Medical Center, Fayetteville, USA; 2 Cardiology, Cape Fear Valley Medical Center, Fayetteville, USA; 3 Cardiovascular Medicine, Creighton University School of Medicine, Omaha, USA

**Keywords:** presyncope, arrythmia, atypical chest pain, cardiac syncope, paroxysmal complete heart block

## Abstract

Paroxysmal complete atrioventricular block (PCAB) is clinically characterized by a sudden change from 1:1 atrioventricular (AV) conduction leading to complete heart block. Patients may have a vast array of symptoms, but commonly, PCAB will lead to syncope and possible sudden cardiac death. The literature currently consists of three different types of PCAB: intrinsic paroxysmal atrioventricular block, extensive vagal paroxysmal atrioventricular block, and extrinsic idiopathic paroxysmal atrioventricular block. Currently, there is no single symptom or sign that is specific to a single type of AV block. PCAB is often missed or overlooked because of its unpredictability and no evidence of conduction disease with a normal 1:1 conduction on routine electrocardiograms. Here, we present a case of a 65-year-old female who has been intermittently symptomatic for four years and was found to have PCAB.

## Introduction

Paroxysmal complete atrioventricular block (PCAB) is clinically characterized by a sudden change from 1:1 atrioventricular (AV) conduction leading to complete heart block. Patients may have a vast array of symptoms, but commonly, PCAB will lead to syncope and possible sudden cardiac death [1]. The literature currently consists of three types of PCAB. Intrinsic paroxysmal atrioventricular block (I-AVB) is due to intrinsic disease of the native AV conduction system. Extensive vagal paroxysmal atrioventricular block (EV-AVB) is due to excessive parasympathetic activation of the native conduction system. EV-AVB has also been called reflex syncope. Extrinsic idiopathic paroxysmal atrioventricular block (EI-AVB) is believed to be associated with but not limited to low levels of endogenous adenosine [2]. PCAB is an important diagnosis to be considered when cardiac syncope is suspected. We present a case of a female who has been experiencing years of atypical chest pain and was incidentally found to have PCAB after a syncopal episode. 

## Case presentation

**Figure 1:**
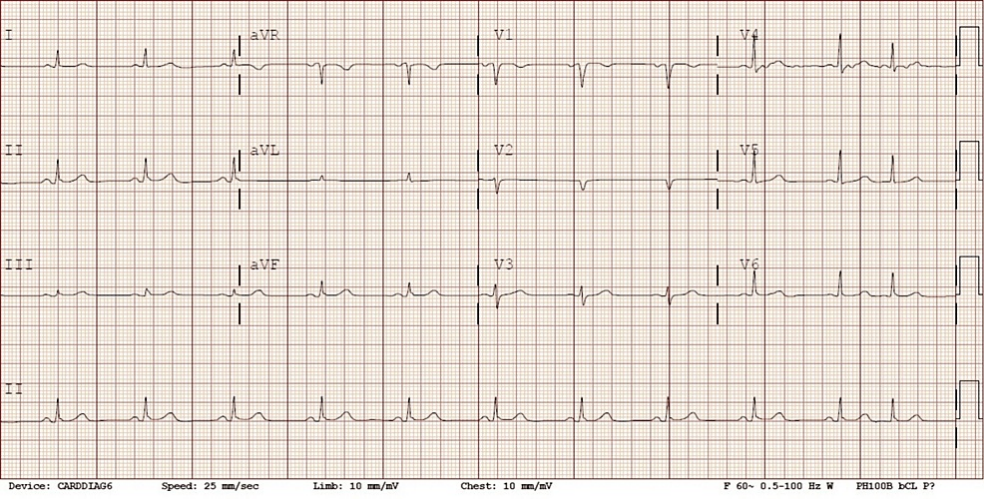
Initial EKG that showed a sinus rhythm rate of 66, atrial premature complex, PR 142, QRSD 89, and QTc 393 EKG: Electrocardiogram

**Figure 2:**
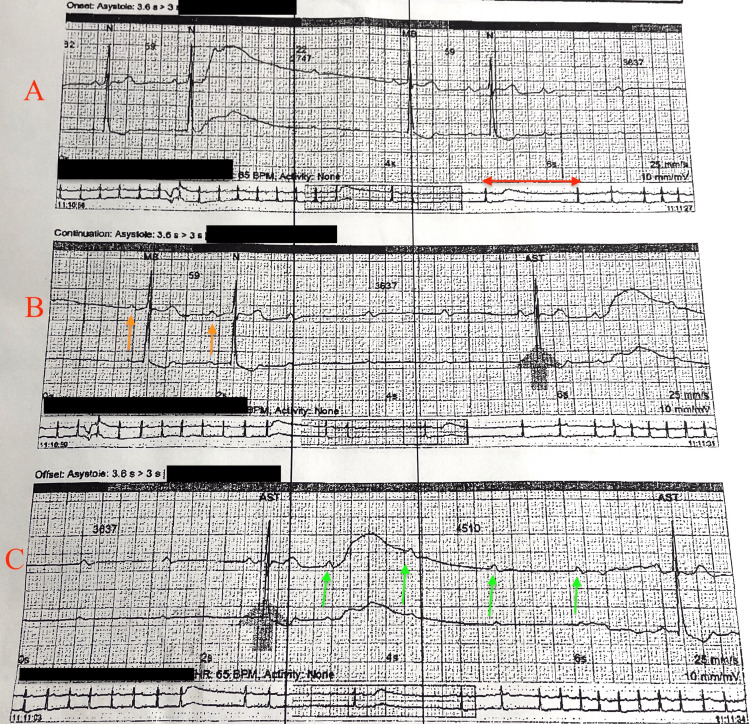
Telemetry strip from a home heart monitor. In rhythm strip A, if you look at the red double-sided arrow you can see the prolonged sinus pauses that were recorded. In rhythm strip B, if you look at the orange single-sided orange arrow, you can appreciate the prolonging P-R interval followed by dropped beats consistent with second-degree AV block Mobitz type 1. In rhythm strip C with the green arrows, you can see consecutive P waves that do not conduct which is suggestive of a high-grade AV block. AV: Atrioventricular

**Figure 3:**
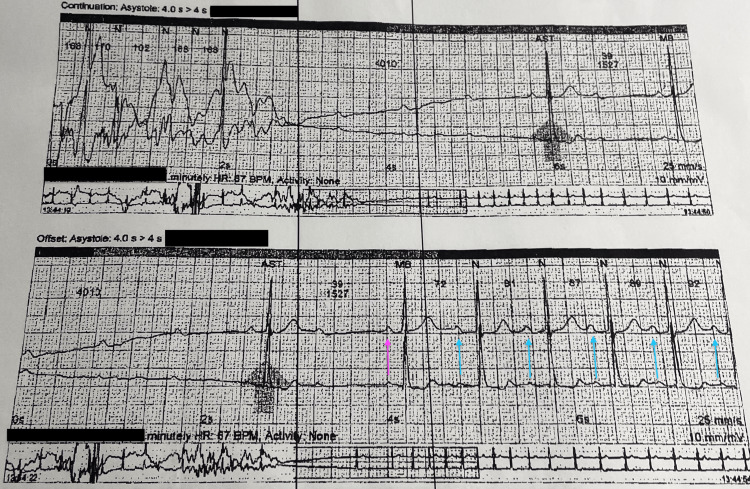
Home heart monitor strips. In the bottom strip you can notice initially that the P waves have no associated QRS. P wave with a QRS is shown by a thick black arrow. The next beat was a P wave with a dropped QRS followed by P waves with associated QRS. You can notice that the pink arrow has a P to R interval shorter than the following P-R intervals. The blue arrows have P-R intervals of the same interval that is longer than the preceding pink arrow consistent with a first-degree AV block. AV: Atrioventricular

A 65-year-old female with a past medical history of hypertension and anxiety presented to the emergency department (ED) as per the request of her primary care provider after an abnormal rhythm was seen on a home cardiac monitor. The patient reported that her symptoms began approximately four years ago when she noticed frequent episodes of chest discomfort which were described as sharp, non-radiating, and intermittent, occurred randomly throughout the day, and lasted 5 to 10 seconds. Nothing exacerbates the symptoms or alleviates the symptoms. The patient does report episodes of dizziness and lightheadedness when she experiences these chest pain episodes. Approximately three years ago, she went to her primary care provider and a stress test was performed, and the results of that stress test were unremarkable. Since then, she has been having approximately three episodes a week but did not reach back out to her primary care provider. The patient states that one month prior to arrival she was walking down her hallway and the next thing she remembers is waking up face down on the floor. Prior to the syncopal episode, she denies any symptoms. At this point, she went back to her primary care provider, and they placed a cardiac monitor on her at approximately 9 AM on the day of arrival. Later that day, when she was having lunch, she was called by the cardiologist's office and told to go to the ED immediately. Upon arriving at the ED, she was asymptomatic, and her electrocardiogram (EKG) showed sinus rhythm (Figure 1). Her cardiac event monitor showed several episodes of asystole after 11:32 AM on the day of arrival, which were up to 4.5 seconds long. The available tracings from these events reveal complete heart block with a feature of first-degree AV block and second-degree AV block Mobitz type 1 (Figures 2, 3). In the ED, telemetry reportedly showed intermittent AV block concerning second-degree type II, but rhythm strips were not available for review. Patient's laboratory values as well as her vitals in the ED were unremarkable. The patient was admitted to the hospital for cardiology evaluation. She remained in sinus rhythm while on floor telemetry, and no further episodes were captured. The patient was evaluated by electrophysiology, and a decision for dual-chamber pacemaker implantation was made. The patient was eventually discharged home with follow-up with cardiology in the following weeks.

## Discussion

PCAB is often missed or overlooked because of its unpredictability, and no evidence of conduction disease with a normal 1:1 conduction on routine EKGs. The exact incidence is unknown due to the paroxysmal nature of the disease. This type of block is seen when repetitive blockade of atrial impulses occurs as they generate toward the ventricle [1]. Our patient was without any known coronary artery disease or any personal or family history of conduction disease, and our patient's underlying mechanism for complete heart block is uncertain. Currently, there is no single symptom or sign that is specific to a single type of AVB [2]. Her presentation and workup have components of two types of extrinsic AV block. EI-AVB has been described as syncope that occurs without prodromes and has been seen with a normal heart structure and normal EKG [3]. I-AVB is usually associated with a baseline bundle branch block, but the presence of a bundle branch block does not exclude an extrinsic type of AV block. Some studies have shown that greater than 40 percent of patients with a bundle branch block has been associated with reflex syncope or EV-AVB [4]. Our patient has components of EV-AVB and EI-AVB PCAB, but it is likely our patient has EI-AVB as opposed to EV-AVB. As seen in Figure 2, our patient has progressive PR prolongation in some leads before progressing to a complete heart block. EI-AVB characteristics that our patient has are narrow QRS, no bundle branch block, sinus rate before AV block, syncopal episode without prodrome, absent structural heart disease, and age over 40 [2]. However, the only characteristic that is consistent with EV-AVB in our patient is the generally progressive PR prolongation that is seen intermittently in some of the home heart monitor strips.

Prolongation of the P-P interval during sinus rhythm, a long pause after premature atrial contraction, or a premature ventricular contraction can cause slow spontaneous depolarization of a diseased His-Purkinje system [1,5,6]. PCAB as defined is always due to disease in the His-Purkinje system and can cause spontaneous phase 4 depolarization when the sodium channels are inactive. Usually after a long pause, the site of the diseased His-Purkinje cells continues to depolarize and become less responsive to continuous impulses [6]. El-Sherif and Jalif performed an unpublished series of 42 paroxysmal AV block cases [5]. Of the 42 cases, 10 underwent a conduction system study; in seven of the conduction system studies, the block was localized in the His bundle. In the remaining three conduction system study cases, the block occurred below the His bundle recording. However, it is not uncommon that intra-His bundle block is underdiagnosed during an electrophysiological study and is labeled instead as infra-His bundle block or even as AV nodal block.

Different treatment modalities exist depending on the underlying type of PCAB. For I-AVB, cardiac pacing will theoretically eliminate syncopal reoccurrence and improve survival [2]. No specific studies have investigated the effect on cardiac pacing in EV-AVB patients. Cardiac pacing is the most effective way to avoid syncope when bradycardia is responsible, but syncope may reoccur in patients who have a vasodepressor reflex in EV-AVB [2]. No trials have been performed to determine the efficiency of cardiac pacing and preventing syncope in EI-AVB. Currently, there have been two small long-term observational cohorts that have shown prevention of syncope in EI-AVB patients [2]. Patients with idiopathic paroxysmal AV block are characterized by low plasma adenosine levels and low expression of A2A adenosine receptors [7]. Two small observational studies for patients with EI-AVB have shown that oral theophylline appears to be effective in preventing syncopal episodes [7,8]. 

## Conclusions

This differential diagnosis is important because I-AVB is a clear indication for permanent pacing while vagally mediated block is not. Our patient had persistent chest pain for years, and although the stress test was unremarkable, a home cardiac monitor was not considered. In a patient with persistent atypical chest pain, it may be reasonable to order a cardiac monitor for surveillance of cardiac arrhythmia. PCAB is a not well-understood entity due to the nature of the disease, but this is a life-threatening condition. We are also aiming to inform clinicians to take a detailed history when PCAB and syncope occur. This will help in determining the correct treatment for the patient because cardiac pacing may not be appropriate for EV-AVB but difficult to determine between the subtypes of PCAB. We hope that this case will help clinicians consider PCAB when the patient presents with atypical chest pain with unremarkable ischemic workup.
